# Menstrual-related symptoms and absence from school among young people in Sweden: a stratified, randomized, population-based survey

**DOI:** 10.1186/s12889-025-24705-w

**Published:** 2025-10-24

**Authors:** Klara Abrahamsson, Eva Åkerman, Sara Ström, Lisa Söderman, Henrik Källberg, Marie Klingberg-Allvin

**Affiliations:** 1 Department of Women’s and Children’s Health, Karolinska Institutet, Stockholm, Sweden; 2 The Public Health Agency of Sweden, Solna, Sweden; 3 Department of Clinical Science and Education, Södersjukhuset University Hospital, Karolinska Institutet, Stockholm, Sweden; 4 Department of Obstetrics and Gynecology, University of British Columbia, Vancouver, Canada

**Keywords:** Menstrual-related symptoms, Dysmenorrhea, Menorrhagia, Premenstrual syndrome, School-related absence, Absenteeism, Young people, Adolescents

## Abstract

**Background:**

Menstrual-related symptoms such as menstrual pain and heavy bleeding impact individuals’ health, quality of life and can limit the ability to engage in daily life activities, including school. Menstrual-related symptoms thus risk reinforcing existing gender inequalities in health among young people, making it an issue of equal rights and public health concerns. No previous study has estimated the prevalence of menstrual-related symptoms and subsequent school absences in Sweden by using population-based data.

**Methods:**

The study aimed to estimate the prevalence of menstrual-related symptoms and school absence among young people aged 16–29 in Sweden, and to examine associations between symptoms, absence, and sociodemographic factors. A sample (*n* = 5,483) of individuals aged 16–29 was drawn from a population-based cross-sectional study which used stratified random sampling. We used logistic regression to test sociodemographic factors associated with school absence due to menstrual-related symptoms.

**Results:**

Menstrual-related symptoms were reported by most of the respondents (91.43%). Menstrual pain was reported by 76.59%, mood changes by 75.70%, ‘other’ menstrual complaints by half (57.88%) and heavy bleeding by 40.14%. Furthermore, 13.70% in total and 19.93% among those aged 16–19 reported that school absence because of menstrual symptoms occurred on every menstruation. Foreign-born individuals and Swedish-born individuals with two foreign-born parents had higher odds of reporting school absence due to menstrual-related symptoms, as did those with parents with short education and those with long-term health issues. ‘Other’ menstrual complaints (such as headache, tiredness and concentration difficulties) had the greatest impact on school absence.

**Discussion:**

Menstrual-related symptoms are widespread among young people in Sweden. The subsequent absence from school is unevenly distributed according to the individual’s origin, parental education and long-term health issues and should be seen as an issue of gender equity and public health concern. Given the importance of schools for learning and development, student health services need to be equipped with screening methods and referral routines. Further studies should focus on socioeconomic inequities in menstrual health, with a particular focus on young migrants and second-generation immigrants.

**Supplementary Information:**

The online version contains supplementary material available at 10.1186/s12889-025-24705-w.

## Background

From both a global [1] and Swedish [2] perspective, the onset of menstruation (i.e., *menarche*) occurs around the age of 12–13, marking a bodily transition from childhood into adulthood. Menstruation is often accompanied by pain and discomfort. Symptoms such as menstrual pain and heavy bleeding impact individuals’ health, quality of life and participation in school and work [3–5]. Menstrual-related symptoms risk reinforcing gender inequalities regarding, for instance, subjective well-being [2], economic status and social engagement [6], making this an issue of equal rights and public health concerns [7]. A recently proposed definition of menstrual health states that menstrual health is an issue for “women, girls, and all other people who experience a menstrual cycle” [8, p. 32]], thus including menstruators who do not identify as women or girls.

Menstrual pain *(dysmenorrhea)* is the most frequently reported menstrual-related symptom. Coexisting symptoms include vomiting, diarrhea, back pain, headache, fatigue and disturbed sleep [9]. An early onset of menstruation, often defined as before 11 or 12 years of age, has been found to be associated with increased levels of menstrual pain [10, 11]. The prevalence of dysmenorrhea worldwide ranges from 45 to 95% [12], and a meta-analysis reported a prevalence of 71% [13]. A Swedish study reported 89% dysmenorrhea among teenagers [5].

Another menstrual-related symptom is heavy menstrual bleeding *(menorrhagia)*, reported by approximately 10–30% of menstruators worldwide [3, 14]. In a previous Swedish study among adolescent girls, 37% reported heavy bleeding [15], which was defined as menstrual blood loss of ≥ 80 mL per cycle [4]. However, because there is a discrepancy between a menstruator’s perceptions and the clinical cut-off, it has more recently been defined as ‘*excessive menstrual blood loss which interferes with the woman’s physical*,* emotional*,* social and material quality of life’* [16, p.6]. Heavy bleeding can have a negative impact on daily life activities, intimate relationships and physical activity [3, 4] and be associated with embarrassment, shame and fear of odor and leakage [17]. Few patients seek medical help for heavy bleeding, and when they do, they tend to be underdiagnosed and poorly treated [3].

Cyclical mood changes related to the menstrual cycle are commonly reported [18]. One Australian study among teenaged girls reported that 73% felt grumpy and 65% reported ‘feeling down or depressed’ before menstruation [19]. For some individuals, symptoms interfere with daily life activities and quality of life [18].

Menstruators seldom seek medical care for menstrual-related symptoms and often delay help-seeking until their symptoms have worsened [6, 19–23]. This could partly be due to a social silence surrounding menstruation, which is rooted in menstrual stigma [17, 24]. Thus, many young menstruators lack knowledge of what is considered a common or expected menstrual experience [22]. Also, studies have shown that menstrual-related symptoms tend to be trivialized among both health professionals and menstruators [12, 25, 26]. These factors may affect the way menstruation is experienced and handled in relation to daily life activities, such as school.

School can strengthen individual health and development, providing young people with education, socialization and a sense of cohesion, which are all central determinants of good health [27]. Menstrual-related symptoms, along with factors like inadequate access to sanitary facilities and pads, as well as feelings of shame and fear of leakage (though not explored in this study), can hinder participation in everyday activities, including attending school [6, 28, 29]. Absence from school because of dysmenorrhea varies from 7.7 to 57.8% worldwide [10]. In previous studies from high-income countries, the prevalence of menstrual-related absence from school among teenagers has varied between 8% and 26% [19, 30]. A recent study among adolescent girls in Sweden reported that 14% had monthly absence from school because of dysmenorrhea [5]. However, no previous study has estimated the prevalence of menstrual-related symptoms and subsequent school absences in Sweden by using population-based data.

### Rationale

Despite being a common and natural part of life, menstruation is often accompanied by symptoms such as pain and heavy bleeding, which can negatively impact health, well-being, and participation in daily activities, including school and work. Menstrual-related symptoms, especially dysmenorrhea and menorrhagia, are prevalent among adolescents and young adults and have been linked to school absenteeism in both global and Swedish contexts. However, stigma, lack of knowledge, and normalization of symptoms may delay care-seeking and limit the ability of menstruators to manage their health effectively. While existing studies have examined menstrual health in smaller samples or specific subgroups, there is a lack of population-based research estimating the prevalence of menstrual-related symptoms and associated school absence among young people in Sweden. Further, there is limited evidence in a Swedish setting on how school absence due to menstrual-related symptoms varies by sociodemographic factors such as origin, parental education, or the presence of long-term illnesses or disabilities. This study seeks to fill that gap and inform public health efforts aimed at improving menstrual health equity.

### Aim

The present study aims to estimate the prevalence of menstrual-related symptoms and subsequent absence from school among young people aged 16–29 years in Sweden and to explore how these outcomes are associated with sociodemographic factors, including gender identity. The specific aims of this study are threefold. First, to estimate the prevalence of menstrual-related symptoms among young people aged 16 to 29 years in Sweden. Second, to assess the prevalence of school absence associated with these symptoms. Third, to examine how menstrual-related symptoms and related school absence are associated with sociodemographic factors, including gender identity, age, origin and parental education level.

## Methods

The current study is based on a cross-sectional survey, UngKAB23, which used stratified random sampling of individuals aged 16–29 years in Sweden. The Public Health Agency of Sweden was responsible for study design of the survey and Statistics Sweden for data collection.

The random sampling of the respondents was carried out on 48 strata based on the following population register variables: sex, age group and seven geographical regions. The inclusion criteria were as follows: being aged 16–29 years, having a Swedish personal security number and comprehending Swedish. The sampling frame consisted of 1,713,499 individuals from the Total Population Registry. 40,000 participants were invited to participate.

**Fig. 1:**
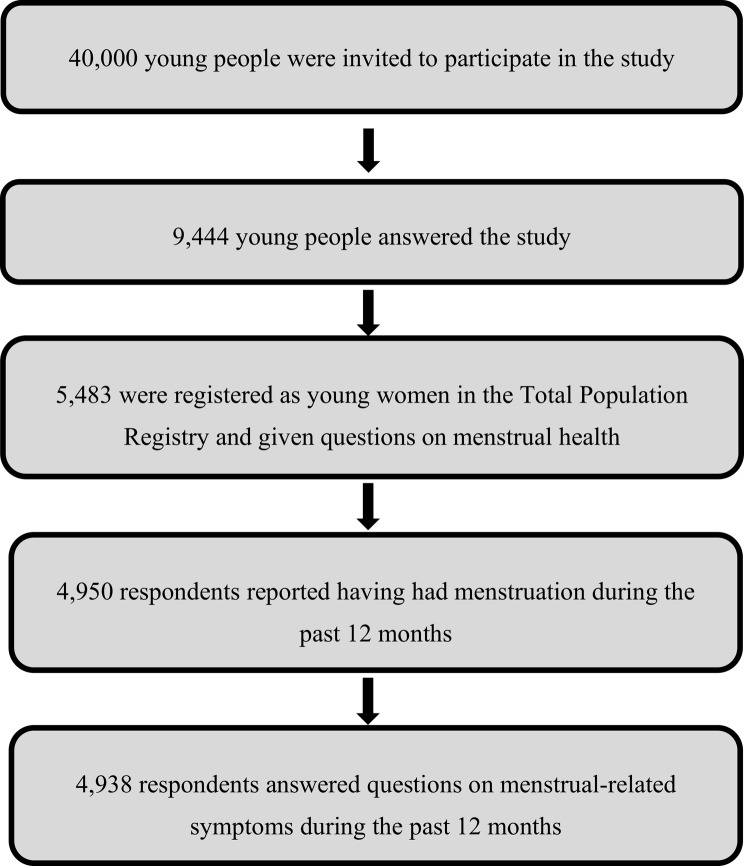
Study flow chart

Data collection was carried out between October 2023 and January 2024. Invitations were sent by email, and two reminders were sent by email, which was followed by an invitation by postal mail and another reminder by postal mail if the respondent had not answered the survey. Out of 40,000 individuals, 9,444 individuals (23.7%) responded, of whom 5,483 (58%) were registered as women in the Total Population Registry; thus, these individuals composed the eligible study population. However, not all individuals coded as women in the Total Population Registry self-identified as women in the survey. By including respondents’ gender identity from the survey data, nonbinary persons and trans men (registered as women) had a chance of being included. To be inclusive of all young people who menstruate, the term “menstruators” is used in this study. Of the 5,483 individuals, 4,950 reported menstruating in the past 12 months, and 4,938 answered follow-up questions on menstrual-related symptoms (Fig. 1).

The questionnaire UngKAB23 contained 46 questions about sexual and reproductive health and rights, and one of several included themes was menstrual health. Menstrual-related symptoms were assessed with the question: *During the past 12 months*,* how often have you experienced: menstrual pain/heavy menstrual bleeding/mood changes before or during menstruation/‘other’ menstrual complaints (e.g.*,* headaches*,* dizziness*,* concentration difficulties*,* tiredness)? Every menstruation/About half the times I have had menstruation/A few times I have had menstruation/Never/Not sure.* Thus, the question did not include duration of symptoms during each menstruation, but rather on how many menstruations each symptom had occurred during the past 12 months. Respondents were allowed to report multiple symptoms. Absence from school was assessed with the question: *During the last 6 months*,* how often have you*,* because of menstrual-related complaints*,* stayed home from school or studies?* Other included variables are described in Appendix 1.

Age (date of birth), country of birth and parents’ country of birth (categorized as: Swedish born, Swedish born with one foreign-born parent, Swedish born with two foreign-born parents, or foreign born) and parents’ years of education (categorized as ≤ 9 years, 10–12 years and ≥ 13 years) were obtained from the Total Population Registry. Information on source of income, including study allowance, during the most recent 12 months was obtained from the LISA registry.

For categorical data, the absolute and relative frequencies are presented, and for normally distributed data, the means and standard deviations. For proportions and odds ratios, we report 95% confidence intervals that consider the calibration weights when relevant. Odds ratios were estimated via logistic regression models. P values less than 0.05 were considered significant.

Multivariate logistic regression, adjusting for age (continuous), origin, parental education, and the existence of any physician-diagnosed long-term illness, disability or long-term health issue, was used to examine associations with school absence due to menstrual-related symptoms. In these analyses, the variable for origin was dichotomized due to similar absence estimates: individuals born in Sweden with one or no foreign-born parent were grouped together. STATA v16.1 was used for analysis.

## Results

The total number of respondents who answered questions on menstrual health (*n* = 5,483) and those who reported having had menstruation in the past 12 months (*n* = 4,950) did not differ by sociodemographic characteristics (Table 1). Among those who had menstruated, 95.45% identified as young women and 4.55% as another gender. Additionally, 12.33% of respondents were foreign born.

**Table 1:** Sociodemographic background among respondents, frequencies and proportions

	All respondents (*n* = 5483)	Respondents who had menstruation within the last 12 months (*n* = 4950)
	*n* (%)	*n* (%)
Gender identity	***n*** **= 5470**	***n*** **= 4947**
Young woman	5209 (95.23)	4722 (95.45)
Other	261 (4.77)	225 (4.55)
Age (years) *	***n*** **= 5483**	***n*** **= 4950**
16–19	1756 (32.03)	1623 (32.79)
20–24	1832 (33.41)	1647 (33.27)
25–29	1895 (34.56)	1680 (33.94)
Origin *	***n*** **= 5454**	***n*** **= 4930**
Swedish born with:		
Swedish-born parents	3961 (72.53)	3548 (71.97)
One foreign-born parent	520 (9.52)	488 (9.90)
Two foreign-born parents	310 (5.68)	286 (5.80)
Foreign born:	670 (12.27)	608 (12.33)
Asia	290	238
EU27 except the Nordic countries	148	136
African region	92	76
Europe except EU27 and the Nordic countries	69	67
Nordic countries except Sweden	55	48
North America	25	23
South America	20	19
Father’s educational level	***n*** ** = 5064**	***n*** **= 4570**
≥ 13 years	2288 (45.18)	2095 (45.84)
10–12 years	2307 (45.56)	2058 (45.03)
≤ 9 years	469 (9.26)	417 (9.12)
Mother’s educational level	***n*** **= 5183**	***n*** **= 4681**
≥ 13 years	3037 (58.60)	2754 (58.83)
10–12 years	1828 (35.27)	1643 (35.10)
≤ 9 years	318 (6.14)	284 (6.07)
Long-lasting health issue	***n*** **= 5473**	***n*** **= 4944**
Yes	1761 (32.18)	1560 (31.55)
No/not sure	3712 (67.82)	3384 (68.45)
Receiving study allowance per age group	***n*** **= 1433**	***n*** **= 1296**
16–19	573 (39.99)	528 (40.74)
20–24	533 (37.19)	478 (36.88)
25–29	327 (22.82)	290 (22.38)

*Parents of the respondents originated from the following regions: Africa, South America, North America, Nordic countries except Sweden, Europe except EU27 and the Nordic countries, EU27 except the Nordic countries, Asia, Oceania and Soviet Union

Nearly 95% of respondents reported to have experienced any menstrual pain (including the option ‘a few times’) during the past 12 months. The most commonly occurring symptoms were menstrual pain and mood changes, reported by 76.59% and 75.70% of respondents, respectively, during every or approximately half of all menstruations during the past 12 months, followed by ‘other’ menstrual complaints (57.87%) and heavy bleeding (40.14%) (Table 2).

**Table 2:** Frequencies and unweighted prevalence rates of menstrual-related symptoms according to respondents’ sociodemographic. *n* = 4938

	Menstrual pain*		Heavy bleeding*		Mood changes*		‘Other’ menstrual complaints*	
	n	% (95% CI)	n	% (95 % CI)	n	% (95 % CI)	n	% (95 % CI)
Total	3788	76.59 (75.39, 77.75)	1984	40.14 (38.79, 41.52)	3743	75.70 (74.48, 76.87)	2858	57.87 (56.44, 59.15)
Gender identity								
Young woman	3600	76.30 (75.10, 77.50)	1876	39.86 (38.50, 41.27)	3572	75.72 (74.48, 76.93)	2727	57.80 (56.39, 59.20)
Other	185	82.22 (76.67, 86.68)	104	46.22 (39.80, 52.77)	170	75.56 (69.51, 80.73)	131	58.22 (51.67, 64.50)
Age group								
16–19	1234	76.08 (73.94, 78.09)	735	45.34 (42.93, 47.78)	1235	76.19 (74.05, 78.20)	929	57.24 (54.82, 59.63)
20–24	1273	77.39 (75.30, 79.34)	649	39.48 (37.14, 41.87)	1239	75.27 (73.13, 77.30)	958	58.24 (55.84, 60.60)
25–29	1281	76.30 (74.20, 78.27)	600	35.78 (33.52, 38.10)	1269	75.63 (73.51, 77.62)	971	57.87 (55.50, 60.21)
Origin								
Swedish born with:								
Swedish born parents	2711	76.43 (75.00, 77.80)	1386	39.11 (38.51, 40.72)	2678	75.52 (74.08, 76.90)	1993	56.20 (54.56, 57.83)
One foreign-born parent	375	77.00 (73.06, 80.52)	204	41.98 (37.66, 46.41)	372	76.39 (72.40, 79.94)	281	57.70 (53.26, 62.01)
Two foreign-born parents	235	82.17 (77.30, 86.18)	150	52.45 (46.66, 58.18)	229	80.07 (75.03, 84.30)	194	68.07 (62.43, 73.22)
Foreign born	458	75.60 (72.00, 78.83)	239	39.44 (35.62, 43.40)	447	73.76 (70.11, 77.11)	380	62.50 (58.58, 66.26)
Mother’s educational level								
≥ 13 years	2124	77.12 (75.52, 78.66)	1089	39.59 (37.78, 41.43)	2071	75.20 (73.56, 76.78)	1541	55.98 (54.11, 57.82)
10–12 years	1253	76.40 (74.29, 78.40)	672	41.00 (38.64, 43.40)	1258	76.75 (74.65, 78.74)	986	60.09 (57.69, 62.43)
≤ 9 years	219	77.11 (71.86, 81.63)	130	45.77 (40.06, 51.60)	215	75.70 (70.38, 80.34)	178	62.90 (57.12, 68.33)
Father’s educational level								
≥ 13 years	1621	77.37 (75.53, 79.12)	825	36.42 (37.34, 41.52)	1581	75.47 (73.58, 77.26)	1173	56.02 (53.88, 58.13)
10–12 years	1555	75.67 (73.77, 77.48)	815	39.68 (37.58, 41.81)	1567	76.29 (74.40, 78.08)	1184	57.59 (55.44, 59.71)
≤ 9 years	340	81.53 (77.52, 84.97)	204	49.04 (44.26, 53.84)	313	75.06 (70.68, 78.98)	274	65.87 (61.17, 70.27)
Age at menarche								
≤ 11	210	84.00 (78.92, 88.04)	133	53.20 (47.00, 59.30)	208	83.20 (78.04, 87.34)	189	75.60 (69.90, 80.52)
12–13	2414	78.02 (76.52, 79.44)	1268	41.01 (39.29, 42.75)	2386	77.14 (75.62, 78.59)	1822	58.89 (57.14, 60.61)
≥ 14	756	74.12 (71.33, 76.71)	383	37.62 (34.70, 40.64)	745	73.04 (70.22, 75.68)	548	53.78 (50.70, 56.82)
Disability								
No	2555	78.88 (76.79, 80.84)	887	38.83 (37.20, 40.48)	2520	74.53 (73.04, 76.00)	1870	55.29 (53.61, 56.96)
Yes	1229	75.55 (74.07, 76.97)	670	43.03 (40.60, 45.50)	1221	78.37 (76.25, 80.34)	984	63.16 (60.73, 65.52)

*Symptoms occurring on every or approximately half of all menstruations during the past 12 months

As also presented in Table 2, heavy bleeding was more commonly reported among 16–19-year-olds than among older individuals. Participants with fathers with ≤ 9 years of education reported higher proportions of symptoms (except mood changes) than those with more educated fathers. All symptoms were most commonly reported by individuals born in Sweden to two foreign-born parents, compared to other origin groups. Individuals who reported having had menarche before the age of 11 reported higher proportions of all symptoms than those with later onset of menstruation. Finally, respondents with any physician-diagnosed illness, disability or long-term health issue reported higher proportions of all symptoms, except menstrual pain. In Supplementary Table 1, the associations between sociodemographic among respondents and menstrual-related symptoms are presented.

Overall, 13.70% of respondents in all ages reported school absences during every menstruation in the past six months, because of menstrual-related symptoms (Table 3). Those with other gender identities than girls reported more absences, as did the youngest age group. Furthermore, foreign-born respondents (25.80%) and those born in Sweden with one foreign-born parent (23.84%) reported school absence more than twice as often as respondents with Swedish-born parents (10.63%). Also, those with parents with ≤ 9 years of education reported approximately twice as much school absence than those with parents with over 12 years of education.

**Table 3:** School absence in the last six months because of menstrual-related symptoms. Frequencies and unweighted proportions

	Every menstruation*		A few times		Never/not sure	
	*n*	% (95% CI)	*n*	% (95% CI)	*n*	% (95% CI)
Total	662	13.70 (12.75, 14.69)	880	18.20 (17.13, 19.30)	3294	68.11 (66.79, 69.41)
Gender identity (*n* = 4833)						
Young woman (*n* = 4617)	615	13.32 (12.37, 14.33)	840	18.19 (17.10, 19.33)	3162	68.50 (67.13, 69.81)
Other (*n* = 216)	45	20.83 (15.92, 26.77)	40	18.52 (14.89, 24.26)	131	60.64 (54.00, 66.94)
Age (years) (*n* = 4836)						
16–19 (*n* = 1601)	319	19.93 (18.03, 21.95)	445	27.80 (25.66, 30.04)	837	52.28 (49.82, 54.72)
20–24 (*n* = 1611)	198	12.29 (10.77, 13.99)	275	17.07 (15.31, 18.99)	1138	70.64 (68.37, 72.81)
25–29 (*n* = 1624)	145	8.93 (7.63, 10.41)	160	9.85 (8.49, 11.40)	1319	81.22 (79.24, 83.04)
Origin (*n* = 4816)						
Swedish born with:						
Swedish-born parents (*n* = 3462)	368	10.63 (9.64, 11.70)	619	17.88 (16.63, 19.19)	2475	71.49 (69.97, 73.00)
One foreign-born parent (*n* = 476)	70	14.71 (11.80, 18.20)	81	17.02 (13.90, 20.66)	325	68.28 (64.00, 72.30)
Two foreign-born parents (*n* = 281)	67	23.84 (19.22, 29.18)	63	22.42 (17.92, 27.66)	151	53.74 (47.88, 59.50)
Foreign born (*n* = 597)	154	25.80 (22.44, 29.46)	113	18.93 (16.00, 22.27)	330	55.28 (51.26, 59.22)
Father’s educational level (*n* = 4464)						
≥ 13 years (*n* = 2056)	236	11.48 (10.17, 12.93)	402	19.55 (17.90, 21.32)	1418	68.97 (66.93, 70.93)
10–12 years (*n* = 2005)	252	12.57 (11.19, 14.09)	338	16.86 (15.28, 18.56)	1415	70.57 (68.54, 72.53)
≤ 9 years (*n* = 403)	81	20.10 (16.47, 24.30)	71	17.62 (14.20, 21.65)	251	62.28 (57.44, 66.89)
Mother’s educational level (*n* = 4574)						
≥ 13 years (*n* = 2701)	329	12.18 (11.00, 13.47)	537	19.88 (18.42, 21.43)	1835	67.94 (66.15, 69.68)
10–12 years (*n* = 1594)	193	12.11 (10.60, 13.80)	264	16.56 (14.81, 18.47)	1137	71.33 (69.06, 73.50)
≤ 9 years (*n* = 279)	72	25.81 (21.01, 31.26)	40	14.34 (10.70, 18.96)	167	59.86 (54.00, 65.46)
Long-term health issue (*n* = 3942)						
Yes (*n* = 1171)	258	17.00 (15.19, 18.97)	290	19.10 (17.20, 21.16)	970	63.90 (61.45, 66.28)
No (*n* = 2771)	401	12.11 (11.04, 13.27)	589	17.78 (16.52, 19.12)	2322	70.11 (68.53, 71.64)
Age at menarche (*n* = 4275)						
≤ 11	47	18.88 (14.48, 24.22)	51	20.48 (15.92, 25.95)	151	60.64 (54.44, 66.52)
12–13	408	13.47 (12.30, 14.73)	564	18.62 (17.27, 20.05)	2057	67.91 (66.23, 69.55)
≥ 14	133	13.34 (11.37, 15.60)	169	16.95 (14.74, 19.41)	695	69.71 (66.78, 72.48)

*Including the options: two days or more on each menstruation/one day each menstruation/one half a day each menstruation

Foreign-born individuals and those born in Sweden with two foreign-born parents had higher odds of absence (including absence ‘a few times’ during the last 6 months) than Swedish-born individuals with one or both parents born in Sweden (Table 4). Having a mother or father with ≤ 9 years of education was associated with increased odds of absence, as was having any long-term health issue. ‘Other’ menstrual complaints showed the strongest association with school absence compared to all other types of symptoms (OR 3.06, 95% CI 2.62, 3.58), followed by menstrual pain (OR 3.01, 95% CI 2.46, 3.67).

**Table 4:** Odds ratios for reporting absence from school because of menstrual-related symptoms.

	Weighted OR (CI 95%)		*p*-value
Gender identity			
Young woman (ref)		1.00	-
Other		1.28 (0.93, 1.77)	0.126
Age (years)			
16-19 (ref)		1.00	
20–24		0.45 (0.39, 0.54)	0.000
25–29		0.28 (0.23, 0.33)	0.000
Origin			
Swedish born (ref)		1.00	-
Swedish born with one foreign-born parent		1.14 (0.91, 1.43)	0.257
Swedish born with two foreign-born parents		2.04 (1.57, 2.67)	0.000
Foreign born		2.17 (1.79, 2.62)	0.000
Father’s educational level			
≥ 13 years (ref)		1.00	-
10–12 years		0.86 (0.75, 1.00)	0.062
≤ 9 years		1.30 (1.02, 1.66)	0.031
Mother’s educational level			
≥ 13 years (ref)		1.00	-
10–12 years		0.79 (0.68, 0.92)	0.002
≤ 9 years		1.52 (1.16, 1.99)	0.002
Long-lasting health issue			
No (ref)		1.00	-
Yes		1.22 (1.05, 1.41)	0.008
Age at menarche			
≤ 11 (ref)			
12–13		0.76 (0.56, 1.03)	0.076
≥ 14		0.72 (0.52, 0.99)	0.046
Menstrual pain			
No (ref)		1.00	-
Yes		3.01 (2.46, 3.67)	0.000
Heavy bleeding			
No (ref)		1.00	-
Yes		2.06 (1.79, 2.37)	0.000
Mood changes			
No (ref)		1.00	-
Yes		2.30 (1.91, 2.78)	0.000
‘Other’ menstrual complaints			
No (ref)		1.00	-
Yes		3.06 (2.62, 3.58)	0.000

In the fully adjusted model, age (continuous), origin, parental educational level and long-term health issues were included, since all these variables were associated with absence from school (Table 5). Foreign-born individuals and those born in Sweden to two parents born abroad had more than twice the odds of reporting absence from school (OR 2.10, 95% CI 1.78, 2.48). In the fully adjusted model, the odds for this group reporting school absence remained nearly two times greater (AOR 1.96, 95% CI 1.58, 2.45). Further, when adding all menstrual-related symptoms in the analyses, the odds for this group changed only slightly, both in the crude model (OR 2.17, 95% CI 1.82, 2.59) and in the fully adjusted model (AOR 1.84, 95% CI 1.45, 2.35). Those reporting any long-term health issue or disability had higher odds of absence in both the crude and adjusted models (AOR 1.55, 95% CI 1.32, 1.81).

**Table 5:** Factors associated with absence from school because of menstrual-related symptoms. Crude and fully adjusted model.

	Crude model	*p*-value	Fully adjusted model*	*p*-value
	OR (95% CI)		OR (95% CI)	
Age (continuous)	0.87 (0.86, 0.89)	0.000	0.85 (0.83, 0.87)	0.000
Origin** (Swedish born and Swedish born with one foreign-born parent = ref)	2.10 (1.78, 2.48)	0.000	1.96 (1.58, 2.45)	0.000
Mother’s educational level (≥ 13 years = ref)	1.06 (0.95, 1.20)	0.284	0.98 (0.85, 1.13)	0.801
Father’s educational level (≥ 13 years = ref)	1.06 (0.95, 1.19)	0.279	1.10 (0.97, 1.25)	0.146
Disability (No = ref)	1.21 (1.05, 1.41)	0.008	1.55 (1.32, 1.81)	0.000

Estimates are presented as weighted odds ratios (OR) with 95% confidence intervals

* Adjusted for age (continuous), origin, mother’s and father’s education level and disability status

** Compared with foreign-born and those born in Sweden with two foreign-born parents

## Discussion

In this first Swedish population-based study that aimed to examine menstrual-related symptoms and subsequent school absence among young people in Sweden, a substantial proportion of respondents reported experiencing menstrual-related symptoms during every menstruation. This finding highlights the pervasive and recurrent nature of these symptoms among young people, with menstrual pain being the most commonly reported symptom.

We also found that the odds of experiencing all menstrual-related symptoms—except mood changes—were higher among individuals with two foreign-born parents compared to individuals with Swedish-born parents. Similarly, individuals who experienced menarche at an earlier age had increased odds of reporting menstrual-related symptoms, and - except for menstrual pain - individuals with a long-term illness or disability.

Furthermore, 13.70% in total and 19.93% among those aged 16–19 reported that school absence because of menstrual symptoms occurred on every menstruation. Foreign-born individuals and Swedish-born individuals to two foreign-born parents had approximately two times higher odds of reporting school absence because of menstrual-related symptoms, in both the crude and fully adjusted regression model. The elevated odds in this group persisted also when including menstrual-related symptoms in the analyses. Further, those reporting any long-term health issue or disability also had higher odds of absence in both the crude and adjusted model. Out of all types of symptoms included, ‘other’ menstrual complaints (such as headache, tiredness and concentration difficulties) had the greatest impact on school absence.

The occurrence of any menstrual pain during the past 12 months (almost 95%) was in line with an Australian study among 13–25-year-olds, which reported 92% [31], but the results of the current study were higher than what was reported in a Swedish study among adolescents in the seventh and eighth grades (80%) [2]. Although the current study does not include measures of pain intensity or experiences of treatment by medical professionals for menstrual pain, previous studies have noted that menstrual pain tends to be normalized and disregarded by medical professionals and that standardized screening methods for menstrual pain are lacking [19, 32]. Thus, more research is needed on the menstrual experiences and symptoms of young people, and menstrual-related symptoms need to be better acknowledged by health professionals.

Our findings show that a greater proportion of individuals born in Sweden to two foreign-born parents reported menarche before or at age 11, than individuals born in Sweden to Swedish parents (11.36% vs. 4.90%). Furthermore, early menarche was associated with a higher frequency of menstrual-related symptoms, consistent with previous reports [2, 10, 11]. Based on this, we suggest that future research should clarify whether early menarche itself, or other associated factors, constitutes a risk factor for menstrual health. There is also a need to examine whether increased amounts of menstrual-related symptoms identified among individuals with two foreign-born parents could be associated with poorer general health status, which in turn could be associated to poor socioeconomic status, as the latter is one of the central determinants of health [27].

That nearly 14% reported menstrual-related school absences on every menstruation is in line with previous Swedish studies [5]. Those with other gender identity than girl reported a higher proportion of absence. Since few respondents identified as a gender other than girl, this result has to be interpreted with caution. However, research on non-binary or transgender menstruators’ experiences of menstruation in school in high income settings is lacking and thus clearly needed in research [33]. It can be suggested that levels of menstrual discomfort and shame might be elevated among those not identifying as young women, since menstruation is closely linked to femininity and female bodily experiences.

It is alarming that almost one fifth (19.93%) of all students in the youngest age group (16–19-year-olds) reported monthly school absence. We argue that this age group, being in the upper secondary school system, need to receive systemized questions on menstrual health and prioritized in future interventions aimed at improving menstrual health. Also, future research should include even younger menstruators than in the current study, since menarche and the years following could be an extra sensitive period for experiencing menstrual discomfort and shame [34]. It could be argued that the need of support in managing menstrual bleeding and associated symptoms is particularly high during the early years following menarche, when individuals are still adapting to these changes.

The finding that menstrual-related school absence was more common among foreign-born respondents, and among those with two parents born abroad, need further attention. This is particularly notable given that their higher odds of absence where not explained by a higher frequency of reported symptoms—since the elevated odds for menstrual-related school absence persisted when controlling for menstrual-related symptoms in the analyses. Future research should explore the underlying reasons for elevated menstrual-related school absenteeism among first- and second-generation immigrant youth, including factors such as their availability of support for menstruation. In Sweden, young people with migration experience often face difficulties accessing sexual and reproductive health services, such as Youth Clinics, mainly due to limited knowledge about availability of care [35]. Whether these barriers also extend to knowledge about services available for menstrual-related symptoms remains to be explored.

It also remains unknown whether restrictive norms—rooted in menstrual stigma and identified as a major contributor to school absence across cultures in low- and middle-income countries [29] —are more prevalent among ethnic minority groups than among ethnic Swedes. This question warrants further investigation through qualitative studies. Another major barrier to school presence in low- and middle-income countries is *period poverty*, which can be broadly defined as a lack of access to and affordability of menstrual-related products, a lack of privacy in hygiene management or a lack of proper education on menstruation [1]. There is evidence of menstrual-related school absence because of a lack of menstrual hygiene products in affluent countries such as the U.S [33, 36]. How prevalent restricting norms and values surrounding menstruation are among Swedish ethnic minority youth, and whether period poverty exists among Swedish youth in general, is unknown. Thus, it is also unknown how this might have an effect on school absence. More research is needed on sociocultural factors concerning menstrual health in high-income settings. Research on menstrual experiences among migrants or refugees in high-income settings is rare [33, 37].

In the present study, those with parents with less years of education were particularly prone to report absence. Parental educational level is an indicator of young people’s socioeconomic status and a central determinant of young people’s health [27]. Regardless of educational level, parents play an important role in young people’s menstrual health, both in terms of the extent to which they tolerate school absence and the extent to which they provide information on and opportunities for conversations about their menstrual health. Because knowledge about menstruation decreases menstrual stigma and increases the ability of menstruators to manage menstruation and participate in daily life activities [29], research on young menstruators’ menstrual health literacy, defined as *‘individual’s access to*,* understanding of*,* assessment and use of information regarding their menstrual health’* [38, p.2], is needed. According to the results of the present study, special attention should be given to menstrual health literacy in relation to socioeconomic factors.

Among our respondents, ‘other’ menstrual complaints, such as headache, tiredness and concentration difficulties, predicted school absence most out of all types of symptoms, closely followed by menstrual pain. Because ‘other’ menstrual complaints lack specificity, further research need to elucidate whether these mainly are physiological symptoms or rather related to social processes such as menstrual-related restrictions, period poverty or shame. The same ambiguity applies to reports of any illness, disability or long-term health issue. There are many possible links between these conditions and menstrual health. For example, research suggests that ADHD, one often highly impairing neurodevelopmental condition among those affected, might be influenced by reproductive hormones, leading to premenstrual mood changes and increasing existing concentration difficulties during menstruation [39].

### Implications for policy and practice

The findings of this study have several implications for policy and practice, particularly within upper secondary schools where 16–19-year-olds study. While the following recommendations are tailored to the Swedish context, many may also be relevant in similar settings.

First, menstrual health should be acknowledged as a possible contributing factor to school absence, and integrated into existing programs and policies targeting absenteeism. Second, previous research shows that the social silencing of menstruation pressures menstruators to hide any signs of it at school, while schools often fail to challenge the perception of menstruation as embarrassing or shameful [11]. To ensure a positive and respectful school environment for all menstruators—aligned with the definition of menstrual health [8]—schools must begin to monitor menstrual-related absence.

In Sweden, school nurses—present in all upper secondary schools—are ideal for addressing menstrual symptoms, assessing their impact on daily life, and identifying students needing support. They can refer students to school doctors or Youth Clinics, staffed by midwives and gynaecologists. Additionally, nurses should inquire about how to create a more menstrual-friendly school environment, supporting attendance and reducing stigma. Interventions may include providing hygiene products, waste bags, and access to water, as well as promoting positive attitudes toward menstruation.

A key component in preventing such negative attitudes, and menstrual stigma, is menstrual health education. Notably, Sweden has offered sexuality education as part of the school curriculum since 1955 and is recognized internationally for having one of the most comprehensive programs [40]. Often, education about menstruation is given within the context of sexuality education. This presents an important opportunity to further strengthen menstrual health education—ensuring it starts early, is age-appropriate, and goes beyond biological and physical aspects. Education should also foster a deeper understanding of what it means to live with and manage menstruation and the whole menstrual cycle, and where to seek care or support if needed.

In summary, this study highlights the need to develop standardized screening for menstrual-related symptoms in Swedish upper secondary schools, as such routines are currently lacking. Previous Swedish research has shown that school nurses seek standardized procedures to better support students with menstrual-related symptoms [25]. To ensure the effectiveness of these procedures, it is essential to involve stakeholders such as youth, school nurses, midwives, and others.

### Methodological strengths and limitations

One limitation of the UngKAB23 study is the low (23.7%) response rate, which limits the generalizability of the results. Because there is a risk of response bias due to systematic patterns of nonresponse among respondents, a nonresponse analysis was performed. Information about nonrespondents was applied when calibrating design weights to reduce the impact of nonresponse patterns. The following factors were associated with a lower likelihood of answering the survey: less years of education, being foreign-born, lower parental education level and low scores in ninth grade. The variables included in the nonresponse analysis were sex, age, country of birth, region, educational level, parental educational level and scores in ninth grade. Thus, the analyses used statistical weights to increase the generalizability of the results to the population of young people in Sweden. Nonetheless, a nonresponse analysis cannot fully eliminate the risk of bias, and response bias remains a concern—particularly with regard to foreign-born youth, who are underrepresented in the data. In the same manner, the confidence intervals for their estimates are wider, indicating greater uncertainty of the estimates.

UngKAB23 is an extensive questionnaire on young people’s sexual and reproductive health, and questions about menstrual health are limited. Due to these limitations, there were no questions about usage of hormonal contraceptives as treatment for menstrual-related symptoms. This limitation likely leads to an underestimation of the actual prevalence of such symptoms.

Another limitation is the absence of questions regarding access to menstrual hygiene products. Including such questions could have offered insights into the prevalence of ‘period poverty’ among Swedish youth.

One factor limiting the depth of the analysis is the broad and heterogeneous categorisation of ‘other’ menstrual complaints, which included both physical and cognitive symptoms such as headaches, fatigue, and concentration difficulties. Dividing these complaints into subgroups could have provided more detailed insights into the specific factors driving menstrual-related school absence. This would have been particularly valuable, given that ‘other’ complaints were stronger predictors of school absence than menstrual pain, heavy bleeding, or mood changes.

One limitation of the registry data is the missing information on parental educational level, affecting 7.64% of respondents for fathers and 5.47% for mothers. These gaps were almost exclusively found among respondents born abroad. Consequently, the influence of parental education on the outcome should be interpreted with caution.

Our data did not contain information on the proportion of students in each age group at the time of data collection. All eligible respondents, regardless of student status, were asked about menstrual health. However, registry data from 2022 revealed that approximately 40% of those receiving study allowances were in the youngest age group (16–19 years), compared to approximately 22% in the oldest (25–29 years) age group. Thus, lower levels of absence were expected among older respondents.

This cross-sectional study does not reflect the complexities that qualitative studies may. For example, it did not measure body image or menstrual stigma and shame. Moreover, the scope of the UngKAB23 questionnaire did not allow for the assessment of perceptions of school belonging or experiences of bullying, both of which are likely to be associated with school absence. Cross-sectional studies also lack the opportunity to investigate potential causal relationships.

## Conclusions

Menstrual-related symptoms are common among young people in Sweden. However, school absence due to these symptoms varies based on origin, parental background, parental educational level and long-term health issues. Since school is vital for young people’s learning and development, absenteeism related to menstruation raises concerns about health equity, gender equality, and public health. It is essential that health professionals recognize and address menstrual-related symptoms. In schools, there is a need to develop standardized screening and referral routines for menstrual-related symptoms, ideally integrated into current student health services.

Future research should explore the interplay between socioeconomic inequities and non-symptom-related factors, such as menstrual shame and stigma, in relation to menstrual health. Particular attention should be given to the experiences of young migrants and second-generation migrants.

## Supplementary Information

Supplementary Material 1.

Supplementary Material 2.

## Data Availability

The data are available from the Public Health Agency of Sweden, but restrictions apply. The data was used under license for the current research and are not publicly available. Data can be made available upon request and with permission of the Public Health Agency of Sweden.
