# Supplementary material for: Menstrual-related symptoms and absence from school among young people in Sweden: a stratified, randomized, population-based survey
Source: BMC Public Health. 2025 Oct 24;25:3602. doi: 10.1186/s12889-025-24705-w (PMC12553222; doi:10.1186/s12889-025-24705-w)
Supplement: Supplementary file 1 — Supplementary Material 1. [file 12889_2025_24705_MOESM1_ESM.docx]

**Supplementary table 1.** Odds of reporting menstrual-related symptoms according to sociodemographic background. Weighted odds ratios (CI) and p-values.

|  | **Menstrual pain*** |  | **Heavy bleeding*** |  | **Mood changes*** |  | | **‘Other’ menstrual complaints*** |  |  |
| --- | --- | --- | --- | --- | --- | --- | --- | --- | --- | --- |
|  | **OR (95 % CI)** | ***p*** | **OR (95 % CI)** | ***p*** | **OR (95 % CI)** | ***p*** | | **OR (95 % CI)** | ***p*** |  |
| **Gender identity** |  |  |  |  |  |  | |  |  |  |
| Young woman (ref) | 1.00 | - | 1.00 | - | 1.00 | - | | 1.00 | - |  |
| Other | 1.39 (0.94, 2.05) | 0.101 | 1.24 (0.92, 1.69) | 0.155 | 0.84 (0.59, 1.19) | 0.326 | | 0.93 (0.69, 1.27) | 0.661 |  |
| **Age (years)** |  |  |  |  |  |  | |  |  |  |
| 16–19 (ref) | 1.00 | - | 1.00 | - | 1.00 | - |  | 1.00 | - | |
| 20–24 | 1.12 (0.93, 1.34) | 0.218 | 0.82 (0.70, 0.96) | 0.013 | 1.07 (0.89, 1.28) | 0.479 | | 1.07 (0.92, 1.26) | 0.356 |  |
| 25–29 | 0.98 (0.82, 1.18) | 0.895 | 0.73 (0.63, 0.86) | 0.000 | 1.03 (0.86, 1.24) | 0.728 | | 1.10 (0.94, 1.29) | 0.219 |  |
| **Origin** |  |  |  |  |  |  | |  |  |  |
| Swedish born with two parents born in Sweden (ref) | 1.00 | - | 1.00 | - | 1.00 | - | | 1.00 | - |  |
| Swedish born with one parent born abroad | 1.09 (0.84, 1.39) | 0.524 | 1.22 (0.98, 1.51) | 0.072 | 1.04 (0.81, 1.22) | 0.771 | | 1.00 (0.81, 1.25) | 0.964 |  |
| Swedish born with two parents born abroad | 1.43 (1.02, 2.02) | 0.038 | 1.72 (1.35, 2.19) | 0.000 | 1.38 (0.99, 1.92) | 0.054 | | 1.49 (1.12, 1.97) | 0.005 |  |
| Foreign born | 0.97 (0.78, 1.21) | 0.804 | 0.97 (0.80, 1.71) | 0.741 | 0.86 (0.70, 1.06) | 0.164 | | 1.16 (0.99, 1.45) | 0.066 |  |
| **Mother’s educational level** |  |  |  |  |  |  | |  |  |  |
| ≥ 13 years (ref) | 1.00 | - | 1.00 | - | 1.00 | - | | 1.00 | - |  |
| 10–12 years | 0.97 (0.83, 1.31) | 0.669 | 1.04 (0.90, 1.19) | 0.560 | 1.05 (0.90, 1.23) | 0.047 | | 1.12 (0.97, 1.28) | 0.098 |  |
| ≤ 9 years | 1.00 (0.73, 1.37) | 0.996 | 1.19 (0.92, 1.55) | 0.186 | 0.97 (0.71, 1.31) | 0.413 | | 1.24 (0.95, 1.63) | 0.114 |  |
| **Father’s educational level** |  |  |  |  |  |  | |  |  |  |
| ≥ 13 years (ref) | 1.00 | - | 1.00 | - | 1.00 | - | | 1.00 | - |  |
| 10–12 years | 0.89 (0.76, 1.04) | 0.107 | 1.02 (0.89, 1.17) | 0.731 | 1.02 (0.88, 1.21) | 0.724 | | 1.00 (0.88, 1.15) | 0.943 |  |
| ≤ 9 years | 1.37 (0.98, 1.92) | 0.068 | 1.45 (1.15, 1.83) | 0.001 | 0.92 (0.71, 1.21) | 0.725 | | 1.42 (1.12, 1.81) | 0.004 |  |
| **Age of menarche** |  |  |  |  |  |  | |  |  |  |
| ≤ 11 (ref) | 1.00 | - | 1.00 | - | 1.00 | - | | 1.00 | - |  |
| 12–13 | 0.65 (0.44, 0.97) | 0.035 | 0.61 (0.45, 0.81) | 0.001 | 0.61 (0.42, 0.89) | 0.011 | | 0.43 (0.31, 0.60) | 0.000 |  |
| ≥ 14 | 0.55 (0.36, 0.83) | 0.005 | 0.51 (0.37, 0.70) | 0.000 | 0.49 (0.32, 0.73) | 0.000 | | 0.34 (0.24, 0.49) | 0.000 |  |
| **Disability** |  |  |  |  |  |  | |  |  |  |
| No (ref) | 1.00 | - | 1.00 | - | 1.00 | - | | 1.00 | - |  |
| Yes | 1.15 (0.98, 1.36) | 0.079 | 1.19 (1.03, 1.37) | 0.012 | 1.24 (1.06, 1.47) | 0.007 | | 1.36 (1.18, 1.56) | 0.000 |  |

*Estimates are presented as weighted odds ratios (OR) with 95 % CI.*

** including symptoms every menstruation or about half of all menstruations during the past 12 months.*
