# Supplementary material for: Menstrual-related symptoms and absence from school among young people in Sweden: a stratified, randomized, population-based survey
Source: BMC Public Health. 2025 Oct 24;25:3602. doi: 10.1186/s12889-025-24705-w (PMC12553222; doi:10.1186/s12889-025-24705-w)
Supplement: Supplementary file 2 — Supplementary Material 2. [file 12889_2025_24705_MOESM2_ESM.docx]

**Appendix 1**. Included variables.

| VARIABLE | QUESTION IN SURVEY |  |
| --- | --- | --- |
| Menstrual-related symptoms | *During the past 12 months, how often have you experienced menstrual pain/heavy menstrual bleeding/mood changes before or during menstruation/‘other’ menstrual complaints (e.g., headaches, dizziness, concentration difficulties, tiredness)?*  *Every menstruation/About half the times I have had menstruation/A few times I have had menstruation/Never/Not sure.* | |
| Absence from school | *During the last 6 months, how often have you, because of menstrual-related complaints, stayed home from school or studies?*  *Two days or more on each menstruation/One day each menstruation/ One half a day each menstruation/A few times during the last 6 months/Never/Not sure.* | |
| Gender identity | *What is your gender identity?*  *Boy/Girl/Nonbinary/I don’t want to categorize myself/I don’t know.*  Data were categorized and named ‘young women’/’other’ in the analyses. | |
| Age at menarche | *When did you have your first menstruation?*  *Not applicable for me/Don’t remember/When I was _ years old (answer in full years)/I have not had my first menstruation yet.* | |
| Long-term health issue | *Do you have any illness, disability or other long-term health issue (e.g., diabetes, allergies, eczema or ADHD) that has been diagnosed by a physician?*  *Yes/No/Not sure.*  ‘No’ was merged with ‘Not sure’ in the analyses. | |
